# Comparison of weighed food record procedures for the reference methods in two validation studies of food frequency questionnaires

**DOI:** 10.1016/j.je.2016.08.008

**Published:** 2017-03-13

**Authors:** Yuri Ishii, Junko Ishihara, Ribeka Takachi, Yurie Shinozawa, Nahomi Imaeda, Chiho Goto, Kenji Wakai, Toshiaki Takahashi, Hiroyasu Iso, Kazutoshi Nakamura, Junta Tanaka, Taichi Shimazu, Taiki Yamaji, Shizuka Sasazuki, Norie Sawada, Motoki Iwasaki, Haruo Mikami, Kiyonori Kuriki, Mariko Naito, Naoko Okamoto, Fumi Kondo, Satoyo Hosono, Naoko Miyagawa, Etsuko Ozaki, Sakurako Katsuura-Kamano, Keizo Ohnaka, Hinako Nanri, Noriko Tsunematsu-Nakahata, Takamasa Kayama, Ayako Kurihara, Shiomi Kojima, Hideo Tanaka, Shoichiro Tsugane

**Affiliations:** a Faculty of Nutritional Science, Sagami Women's University, Kanagawa, Japan; b Faculty Division of Human Life and Environmental Sciences, Research Group of Food Science and Nutrition, Nara Women's University, Nara, Japan; c Epidemiology and Prevention Group, Research Center for Cancer Prevention and Screening, National Cancer Center, Tokyo, Japan; d Department of Food Science and Nutrition, Faculty of Human Life and Environmental Sciences, Nagoya Women's University, Aichi, Japan; e Department of Health and Nutrition, School of Health and Human Life, Nagoya Bunri University, Aichi, Japan; f Department of Preventive Medicine, Nagoya University Graduate School of Medicine, Aichi, Japan; g Department of Cardiology, Hiraka General Hospital, Akita, Japan; h Public Health, Department of Social and Environmental Medicine, Graduate School of Medicine, Osaka University, Osaka, Japan; i Department of Community Preventive Medicine, Niigata University Graduate School of Medical and Dental Sciences, Niigata, Japan; j Department of Health Promotion Medicine, Niigata University Graduate School of Medical and Dental Sciences, Niigata, Japan; k Division of Cancer Registry, Prevention and Epidemiology, Chiba Cancer Center Research Institute, Chiba, Japan; l Laboratory of Public Health, School of Food and Nutritional Sciences, University of Shizuoka, Shizuoka, Japan; m Department of Public Health, Nagoya City University Graduate School of Medical Sciences, Aichi, Japan; n Division of Epidemiology and Prevention, Aichi Cancer Center Research Institute, Aichi, Japan; o Department of Public Health, Shiga University of Medical Science, Shiga, Japan; p Department of Epidemiology for Community Health and Medicine, Kyoto Prefectural University of Medicine, Kyoto, Japan; q Department of Preventive Medicine, Institute of Biomedical Sciences, Tokushima University Graduate School, Tokushima, Japan; r Department of Geriatric Medicine, Graduate School of Medical Sciences, Kyushu University, Fukuoka, Japan; s Department of Public Health, Showa University School of Medicine, Tokyo, Japan; t Department of Community Medicine Management, Faculty of Medicine, Shimane University, Shimane, Japan; u Department of Advanced Cancer Science, Yamagata University Faculty of Medicine, Yamagata, Japan; v Department of Preventive Medicine and Public Health, School of Medicine, Keio University, Tokyo, Japan; w Faculty of Health Promotional Science, Tokoha University, Shizuoka, Japan; x Department of Epidemiology, Nagoya University Graduate School of Medicine, Aichi, Japan

**Keywords:** Dietary assessment method, Dietary records, Standardization

## Abstract

**Background:**

Although open-ended dietary assessment methods, such as weighed food records (WFRs), are generally considered to be comparable, differences between procedures may influence outcome when WFRs are conducted independently. In this paper, we assess the procedures of WFRs in two studies to describe their dietary assessment procedures and compare the subsequent outcomes.

**Methods:**

WFRs of 12 days (3 days for four seasons) were conducted as reference methods for intake data, in accordance with the study protocol, among a subsample of participants of two large cohort studies. We compared the WFR procedures descriptively. We also compared some dietary intake variables, such as the frequency of foods and dishes and contributing foods, to determine whether there were differences in the portion size distribution and intra- and inter-individual variation in nutrient intakes caused by the difference in procedures.

**Results:**

General procedures of the dietary records were conducted in accordance with the National Health and Nutrition Survey and were the same for both studies. Differences were seen in 1) selection of multiple days (non-consecutive days versus consecutive days); and 2) survey sheet recording method (individual versus family participation). However, the foods contributing to intake of energy and selected nutrients, the portion size distribution, and intra- and inter-individual variation in nutrient intakes were similar between the two studies.

**Conclusion:**

Our comparison of WFR procedures in two independent studies revealed several differences. Notwithstanding these procedural differences, however, the subsequent outcomes were similar.

## Highlights

• The procedures of weighed food records in two independent studies were compared.
• General procedures of the dietary records were the mostly same for both studies.
• Differences were found in the setting of survey days and the survey sheet.
• Setting of survey days did not influence the intra- and inter-individual variation.
• Differences in survey sheets did not influence the portion sizes.

## Introduction

Pooled analysis of large-scale cohort data from various populations has the advantage of producing more robust evidence for even small effects of diet on disease than a single study can provide.^1^, ^2^, ^3^, ^4^, ^5^, ^6^ Cohort studies commonly evaluate dietary intake exposure in individual subjects using a specific food frequency questionnaire (FFQ).^7^, ^8^, ^9^ However, pooling dietary intake of foods and nutrients estimated using different FFQs needs to be done carefully because absolute intake levels may not be comparable between different FFQs.

The Japan Multi-Institutional Collaborative Cohort Study (J-MICC Study) and the Japan Public Health Center-based Prospective Study for the Next Generation (JPHC-NEXT Study) are large prospective cohort studies that aim to investigate diet-disease associations. Both studies have adopted FFQs that are most suited for their respective study populations. We, the two study groups, are now going to calibrate the dietary intakes estimated using the FFQs between the studies based on the validation studies using weighed food record (WFR) methods as the reference method.

Open-ended dietary assessment methods, which measure what a subject actually ate during a certain period of time, including WFRs, are considered more comparable than FFQs. However, because the WFRs of the two studies were conducted independently, it is necessary to compare procedures and some results to determine whether the estimated intake using WFR of the two studies can be combined for the integrated analysis for calibration.

In this paper, we assessed the procedures of the WFRs used in the two studies to describe the procedures of dietary assessment and compared the subsequent outcomes. The steps of the procedures were selected based on details that were usually listed in the standard procedures of the research dietitians but were sometimes unwritten. We also described the design of the validation study of the FFQ for the two large cohort studies. This study may provide suggestions for the pooling of WFR data derived from two or more surveys.

## Methods

### Subject areas and study period in the main studies

The J-MICC study is a multicenter cohort study investigating the relation between lifestyle, genetic factors, and diseases, such as cancers. It includes community-based cohorts and medical facility-visitor cohorts (medical checkup and medical consultation). The age of subjects was 35–69 years old at baseline.^10^

The JPHC-NEXT study is a large-scale cohort study for local residents that aims to investigate the relation between lifestyle/living environment and diseases. Subjects are residents of the study areas (available at http://epi.ncc.go.jp/jphcnext/area/index.html) aged 40–74 years at baseline.

### Weighed food records

Since the two studies used different FFQs to assess the diet of their cohorts, the comparability of these FFQs needs to be evaluated using the WFR of the validation studies. This methodology is a “common method” in validation studies of questionnaire integration.

For the J-MICC study, we selected 133 people who provided informed consent from five of the main study areas in which data collection was complete as of March 15, 2014 (Aichi Cancer Center [ACC], Shizuoka, Takashima [Shiga Prefecture], Saga, and Tokushima). Of these, 128 people completed the scheduled survey period, whereas five dropped out due to illness or business or housework pressures. Participation was on an individual rather than family basis. The survey period for an individual was basically 1 year. The start of the survey ranged from October 2011 in the Saga area to May 2012 in the Shiga area. All subject replied to questionnaires at the beginning and end of the study. Dietary assessment was conducted using a WFR in accordance with the methods of the National Health and Nutrition Survey^11^ for a total of 12 days, consisting of 3 days separated by 1-day intervals, and including 1 weekend day, in each of the four seasons. All subjects were given a full explanation of the study, and provided (or loaned) the digital scales required to weigh foods before the study start. Subjects were asked to list all foods and drinks (excluding drinking water) consumed over a certain period of time on the survey sheet. In addition, before intake, the subjects were asked to place foods and drinks on the distributed paper mat (with a checkered pattern to illustrate serving size) and take photos. Recording was based on weight, but serving size recording was also acceptable. After completion of the survey period in each season, the subjects were asked to mail the survey sheet to the regional study director. All contents were confirmed, and additional information was collected from the subjects via telephone or e-mail. Some subjects were given a face-to-face interview at the time of the first survey. Photos were secondarily used at the time of weight estimation, at which time further information was collected. All confirmation of details, weight conversion of recorded serving sizes, food coding, and data input were overseen by trained investigators. Input was performed using an assisted input interface developed in Microsoft Excel (Microsoft Corp, Redmond, WA, USA) which was analogous to the interface of the National Health and Nutrition Survey.^11^ A processed food/seasoning code and dining-out/prepared food code were used only if no codes were listed in the Standard Tables of Food Composition in Japan Fifth Revised edition (STFC5rev).^12^ These codes are used in the National Health and Nutrition Survey^11^ of the public (available at http://www0.nih.go.jp/eiken/nns/system/data.html). Foods and dishes that did not fall into the above categories were converted into foods listed in the STFC5rev^12^ by referencing foods or combinations containing similar nutrients. Intake calculation was done using the STFC5rev.^12^

For the JPHC-NEXT study,^13^ five of the areas participating in the main and cooperating cohort studies (Yokote [Akita Prefecture], Saku [Nagano Prefecture], Chikusei [Ibaraki Prefecture], and Murakami and Uonuma [Niigata Prefecture]) were designated as subject areas and recruitment was started. A total of 255 subjects aged 35–81 years who provided informed consent were included in the assessment. Participation on a family-unit basis was encouraged, but individual participation was acceptable. Two subjects who moved away from the study area were excluded, leaving 253 subjects who completed the study. The survey period was approximately 1 year, between November 2012 and November 2013. In addition to blood sampling, 24-h urine collection, and questionnaire responses, a diet survey was conducted using WFR in accordance with the methods of National Health and Nutrition Survey^11^ for a total of 12 days, consisting of 3 consecutive days, including 1 weekend day, in each of the four seasons. The subjects were asked to participate in an orientation before initiation. Manuals and instruments necessary for the survey were provided, and the weighing and entry methods were explained. The subjects were asked to list all foods and drinks consumed over a certain period of time on a survey sheet. Weight was recorded; otherwise, serving size was recorded when food size was fixed, such as with sliced bread and eggs, or when weighing was impossible, such as when dining-out or eating ready-made meals. On the day after the last day, a face-to-face interview was conducted to confirm the list and collect additional information. The subjects were not asked to take food photos, but when they voluntarily did take photos and brought them to the interview, these was used as auxiliary tools to estimate weight. When a subject was unable to visit an office, the original survey sheet was sent via mail or fax, and the confirmation interview was done via telephone. Trained investigators were in charge of face-to-face interviews, weight conversion of recorded serving sizes, food coding, and input. Weight estimation was performed during the interview using auxiliary tools for weight estimation. The software “shokuji-shirabe” (developed by the National Institute of Biomedical Innovation, Health and Nutrition, Tokyo, Japan; available at http://www0.nih.go.jp/eiken/chosa/kenkoeiyo.html) was used in accordance with the National Health and Nutrition Survey^11^ following approval for “Utilization Other Than for Intended Purposes”. Processed food codes and dining-out/prepared food codes not listed in the Standard Tables of Food Composition in Japan 2010 (STFC2010)^14^ and the proportional distribution function for households in the software “shokuji-shirabe” were used. When foods and dishes that did not fall into the above categories were used, these were converted into foods listed in the STFC2010^14^ on the basis of foods or combinations containing similar nutrients. Intake calculation was done using the STFC2010.^14^

We show the data collection sequence of the two studies described above in Table 1.

**Table 1:** Data collection in validation studies of the J-MICC and JPHC-NEXT studies.

J-MICC							
	FFQ1^a^	WFR1^b^	WFR2^b^	WFR3^b^	WFR4^b^	FFQ2^a^	JPHC NEXT FFQ^a^
Saga	2011, Sep	Oct	2012, Feb	May	Aug	Oct	Nov
ACC	2012, Jan	Feb	May	Aug	Nov	2013, Jan	Feb
Tokushima	2012, Feb	Mar	June	Sept	Dec	2013, Feb	Mar
Shizuoka 1	2012, Feb	Mar	June	Sept	Dec	2013, Feb	Mar
Shizuoka 2	2013, Feb	Mar	June	Sept	Dec	2014, Feb	Mar
Takashima	2012, Apr	May	Aug	Nov	2013, Feb	Apr	May

JPHC-NEXT								
	FFQ1^a^	J-MICC FFQ1^a^	WFR1^b^	WFR2^b^	WFR3^b^	WFR4^b^	FFQ2^a^	J-MICC FFQ2^a^
Yokote	2012, Nov	Nov	Nov, Dec	2013, Feb, Mar	May	Aug	Nov	Nov
Saku	2012, Dec	Dec	Dec	2013, Mar	June	Oct	Dec	Dec
Chikusei	2012, Nov	Nov	Nov	2013, Feb	May	Sept	Nov	Nov
Murakami	2012, Nov	Nov	Nov, Dec	2013, Mar	June	Oct	Dec	Dec
Uonuma	2012, Oct	Nov	Nov	2013, Feb	May	Aug	Dec	Dec

J-MICC, The Japan Multi-Institutional Collaborative Cohort Study; JPHC-NEXT, The Japan Public Health Center-based Prospective Study for the Next Generation; FFQ, food frequency questionnaire; WFR, weighed food record.

a FFQ1 or 2: original FFQ of the respective studies at first or second use.

b WFR1to 4: WFRs for first to fourth use.

To facilitate standardization in the JPHC-NEXT study, staff in charge of dietary assessment in the J-MICC and JPHC-NEXT studies met prior to initiation of the JPHC-NEXT study to discuss dietary assessment standardization. The two studies were planned and performed independently, and reconciliation of some items was difficult. Nevertheless, the method of the J-MICC study was followed wherever possible. With regard to procedures for WFR in the two protocols, details of preparation, general matters, and implementation were described and compared item by item.

For the present study, the protocols of the J-MICC and JPHC-NEXT questionnaire validation studies were approved by the ethical review boards of Aichi Cancer Center, Aichi, Japan, and the National Cancer Center, Tokyo, Japan before the study was started.

### Statistical analysis

As basic items, the 12-day data for WFRs collected in each study were analyzed for arithmetic mean values and standard deviation of the number of food items and dishes per person per day. In addition, the cumulative percentages of foods that contributed energy and major nutrients (carbohydrate, protein, lipid, sodium, and potassium) were calculated. For food items common to the two FFQs or major items of meats, vegetables, and seasonings, the portion size distribution of 11 items (three meats, five vegetables, and three seasonings) was investigated separately according to sex to confirm the influence of differences in survey sheet recording method (individual versus family participation). The relative contributions of intra-individual and inter-individual variance in nutrient intake were calculated, and intra-individual and inter-individual variance ratios and intra-individual variance ratios between the two studies were derived to confirm the influence of the difference in selection of multiple days (non-consecutive versus consecutive days). The number of food items and dishes, cumulative percentages of food product contribution for energy and major nutrients, and the portion size distribution of 11 items were also calculated for subjects aged 35–69 years old, the specified age limit of subjects in the J-MICC study. Analysis of variance was performed using SAS software (version 9.3; SAS Institute Inc, Cary, NC, USA).

## Results

Percentages of men subjects in the J-MICC and JPHC-NEXT studies were 51.6% and 42.3%, respectively. Mean (standard deviation [SD]) ages of men in the J-MICC and JPHC-NEXT studies at baseline were 52.0 (SD, 8.6) years and 56.9 (SD, 9.9) years, respectively. Mean (SD) energy intakes of men in the two studies were 2259 (SD, 357) kcal and 2297 (SD, 450) kcal, respectively. Mean (SD) ages of women were 52.3 (SD, 7.7) years and 56.0 (SD, 9.1) years, and mean (SD) energy intakes were 1777 (SD, 316) kcal and 1797 (SD, 309) kcal, respectively.

Table 2 compares the procedures used in the J-MICC and JPHC-NEXT studies for weighed food records and standardization. Although the procedures used in the dietary assessments were independent, the two methods were almost completely consistent. Specifically, points in common included 1) the surveys were conducted using WFR (with serving size recording in some cases); 2) investigators were trained to improve survey standardization performance; 3) details were confirmed with subjects through face-to-face or telephone interview; 4) food models were used as auxiliary tools to estimate weight (for 11 ingredients; ingredients were similar to those in the J-MICC study); and 5) foods and supplements that did not require recording in the record sheets and the correspondence of these were the same.

**Table 2:** Comparison of procedures for weighed food records and standardization (J-MICC versus JPHC-NEXT).

Procedure		J-MICC (as of March 15, 2014, limited to survey subject area)	JPHC-NEXT
Preparation	Training to improve investigator performance in survey standardization	Subjects: - Registered dieticians (researchers at universities and research institutes, graduate students, and part-timers) - Number of investigators and skill level: 17 people: minimum number experienced with intake recording: ≥60% Standardization training: - Training of all investigators by the Central Office (except 2 people handled by local offices) - Studying the rules of nutrition measurement using uniform manuals and materials, and weight estimation proficiency test by an e-learning control system Standardization test (1–3 for all investigators) Coding-related contents: - Coding (particularly seasonings) - Weight conversion	Subjects: - Registered dieticians, dieticians, students (researchers at universities and research institutes, graduate students, and local part-timers) - Number of investigators and skill level: 35 people; minimum number experienced with intake recording: ≥60% Standardization training: - Training of all investigators by the Central Office (approximately 1 day in total) - Studying the survey procedure based on uniform manuals and materials, and practice role-play using uniform questions and answers Retraining and standardization test (6 months later, for all investigators) - Coding - Weight conversion - Hearing
General	Subjects	35- to 69-year-old males and females: 132 people; dropouts: 5 people Individuals	35- to 81-year-old males and females: 255 people; dropouts: 2 people Individuals (proportional distribution by members of family)
	Subject areas (Number of offices)	5 study cohorts: Aichi Cancer Center (Aichi Prefecture), Shizuoka, Takashima (Shiga Prefecture), Saga, Tokushima 1 central office: Central Office (Aichi)	5 study cohorts: Yokote (Akita Prefecture), Saku (Nagano Prefecture), Chikusei (Ibaraki Prefecture), Murakami and Uonuma (Niigata Prefecture) 3 competent offices Central Office (Yokote and Saku), Niigata Office (Murakami and Uonuma), Chikusei Office
	Method of recording the intake and confirming data	3 alternating days (every other day, including 1 weekend day) × 4 seasons Details confirmed by phone or e-mail so that the subjects can take notes (in some cases, at interview on the first occasion	3 consecutive days (including 1 weekend day) × 4 seasons Details confirmed by face-to-face interview the day after the last day of intake recording (or in some cases, by phone after mail or faxing of the original sheet)
	Process and division of roles	Interview, weight conversion, input, and collation: Investigators or local offices Data cleaning: Central Office	Interview and weight conversion: Investigators Input and collation: Local offices Data cleaning: Central Office
	Survey sheet	Survey sheet per person 1 recording form per meal (breakfast, lunch, dinner, others)	Conforms to National Nutrition Survey (proportional distribution by member of family) 1 recording form per meal (breakfast, lunch, dinner, snack)
Recording	Auxiliary tools for weight estimation	Portion size recording (in some cases, serving size recording) (1) Photo (2) Food model: 11 ingredients* (3) Containers used as standard tableware	Portion size recording (in some cases, serving size recording) (1) Food model: 11 ingredients* (2) Booklet showing real-scale foods and dishes (3) Cards showing real-scale dishes (4) Used containers as standard tableware
	*Ingredients of food model	Rice (140 g, 220 g, 250 g), sliced bread, carrot, spinach (boiled), horse mackerel (cut open and dried), flatfish (fillet), mackerel (fillet), sliced pork (leg), chicken (leg without skin)	
	Confirmation manual (items to be confirmed at interview)	(1) Weight (serving size) (2) proportion (proportional distribution by member of family) or weight (total and individual) (3) with or without skin, disposal (4) raw, dried, or cooked (at measurement) (5) kinds and parts (6) omitted seasonings (cooking oil, etc.) (7) leftovers (broth, stock, noodle soup, etc.) (8) any incomplete entry	
	Targets for interview portion	Subjects of the survey, in principle (in some cases, persons in charge of cooking)	Subjects of the survey, in principle (substituted by person in charge of cooking in cases of subject absence)
	Items not to be recorded, exceptions, etc.	Supplements: not recorded Enriched foods and specified health foods: Substituted with common foods Water content: Broth, stock, noodle soup, and water for dilution to be recorded, drinking water not to be recorded	Supplements: not recorded Enriched food and specified health foods: substituted with common foods Water content: recorded
Coding		Conforms to National Health and Nutrition Survey (using dining-out and prepared food database, processed food database, and guides) Cases of difficult coding to be shared among all regions (using web message boards and Google Drive)	
Input		Assisted input interface using MS Excel	Conforms to National Health and Nutrition Survey (using the proportional distribution by member of family function of “shokuji-shirabe”)
Data cleaning		Data accumulated in the input error search database, error extraction, and data cleaning	
Intake calculation		Standard Table of Food Composition in Japan, Fifth Revised edition	Standard Table of Food Composition in Japan 2010

J-MICC, The Japan Multi-Institutional Collaborative Cohort Study; JPHC-NEXT, The Japan Public Health Center-based Prospective Study for the Next Generation.

In contrast, several differences were identified, including 1) setting of the survey dates; 2) survey sheet recording method (individual versus family participation); and 3) method of determining fluid intake. With regard to 1), the J-MICC study used recording on 3 non-consecutive days, with a 1-day period between each recording day, whereas the JPHC-NEXT study used recording on 3 consecutive days (both including 1 weekend day). Regarding 2), the J-MICC study basically followed the individual subject, whereas the JPHC-NEXT study also allowed the recording of subjects as family units (e.g., a couple). In this method, subjects listed family intake in the survey sheet, and individual intakes were determined using proportional distributions reported by the subject. Finally, regarding 3), the J-MICC study recorded broth, stock, noodle soup, and water used for dilution, but not drinking water, whereas the JPHC-NEXT study also recorded drinking water. Accordingly, we excluded data for drinking water from the JPHC-NEXT study to ensure the use of common data for intake calculation.

From the 12-day WFR data, the number of food items and dishes per person per day were calculated. The number of food items in the J-MICC and JPHC-NEXT studies was 56.0 (SD, 13.1) and 58.3 (SD, 14.9), and the number of dishes was 16.6 (SD, 3.4) and 17.8 (SD, 4.4), respectively. Calculated cumulative percentages of food product contributions to energy and major nutrients (carbohydrate, protein, lipid, sodium, and potassium) are shown in eTables 1–6. The top five food products contributing energy were matched in the two studies, although their order differed. Of the top five food products for other major nutrients, at least three of five matched, again in random order. In addition, the cumulative percentages of contribution to the top 30 food products for all nutrients other than potassium shown in the tables were slightly higher in the J-MICC than in the JPHC-NEXT study: 57.5% versus 54.5% for energy, 69.5% versus 66.5% for carbohydrate, 51.6% versus 50.6% for protein, 65.5% versus 60.5% for lipid, and 74.2% versus 69.3% for sodium, respectively. In contrast, this variable was higher in the JPHC-NEXT (48.0%) than in the J-MICC study (47.5%) for potassium only.

Among food items common to the two FFQs or major items, Table 3 shows the portion size distribution of 11 items by sex. Comparison of medians between the J-MICC and JPHC-NEXT studies indicated percent differences of 2.8%–20.9% (men) and −10.0% to −6.5% (women) for three meat items, −29.6% to 0% (men) and −23.5% to 3.6% (women) for vegetables, and 3.8%–25.0% (men) and 7.4%–25.0% (women) for three seasoning items, respectively.

**Table 3:** Distribution of portion size according to food group using dietary record for 12 days.

Food group	J-MICC					JPHC-NEXT					% Difference^a^
	Frequency of appearance (times/person)	25th percentile (g/time)	Median (g/time)	75th percentile (g/time)	Inter quartile range (g/time)	Frequency of appearance (times/person)	25th percentile (g/time)	Median (g/time)	75th percentile (g/time)	Inter quartile range (g/time)	
**Men**	**n** = **66**					**n** = **107**					
Beef	5	14.3	30.5	60	45.7	3	8.3	24.1	50.8	42.6	20.9
Pork	11	12	30	52.8	40.8	11	12.9	29.2	51.9	39	2.8
Chicken meat	7	16.8	35	61.8	45	5	18.1	34	65	46.9	2.8
Pumpkin	2	21.5	40	65.7	44.1	2	25	40	63.9	38.9	0
Carrot	17	5	10	18.9	13.9	15	5	10	17	12	0
Broccoli	3	14.5	22.8	36.4	21.9	2	15	25	41.6	26.6	−9.9
Cabbage	9	11	27	50	39	9	18.3	35	60	41.7	−29.6
Japanese radish	7	15	30	53	38	8	20	37.5	65	45	−25
Soy-sauce	39	2	3.1	6	4	32	1.8	3	6	4.3	3.8
Salt	36	0.2	0.4	0.7	0.5	37	0.2	0.3	0.6	0.5	25
Soybean paste	11	5.6	9	12	6.4	15	5	8.6	12	7	4.2
**Women**	**n** = **62**					**n** = **146**					
Beef	4	13.7	28.2	48.5	34.8	2	11.5	30	53.4	41.9	−6.5
Pork	8	12.5	25	46	33.6	9	12.7	27.5	50	37.3	−10
Chicken meat	6	19	30	50	31	5	17.5	32.8	54.6	37.1	−9.3
Pumpkin	2	20	35.1	62	42	3	25	41.3	69.7	44.7	−17.6
Carrot	15	5.8	10	20	14.3	14	5	9.6	15	10	3.6
Broccoli	2	18.2	27	36.4	18.2	3	18.2	27.3	44	25.8	−1.1
Cabbage	8	15.5	30	50	34.5	8	18	33.9	60	42	−12.9
Japanese radish	6	20	30	55	35	8	20	37.1	65	45	−23.5
Soy-sauce	33	2	3.4	6	4	29	1.6	3	6	4.4	12.9
Salt	28	0.2	0.4	0.8	0.6	32	0.1	0.3	0.6	0.5	25
Soybean paste	10	5.6	9	11.8	6.2	14	5	8.3	11.3	6.3	7.4

J-MICC, The Japan Multi-Institutional Collaborative Cohort Study; JPHC-NEXT, The Japan Public Health Center-based Prospective Study for the Next Generation.

When calculated within the limits of definition age of the J-MICC study, the results were similar (data not shown).

a % Difference: (J-MICC median − JPHC-NEXT median)/J-MICC median * 100.

When limited to subjects aged 35–69 years, the specified age of subjects in the J-MICC study, the number of food items and dishes, cumulative percentages of food product contribution for energy and major nutrients, and the portion size distribution of 11 items were all closely similar (data not shown).

The relative contributions of intra-individual and inter-individual variance in nutrient intake were calculated, and intra-individual and inter-individual variance ratios were determined. The intra-individual variance ratios between the two studies were also determined (Table 4). Percentage contributions of intra-individual variance in the J-MICC and JPHC-NEXT studies ranged from 41.2% and 43.5% (for energy) to 92.7% and 92.0% (for retinol activity equivalents), respectively. A greater contribution of intra-individual variance compared with inter-individual variance was shown for more than 18 of 23 calculated items in both studies. The highest intra-individual and inter-individual variance ratio was seen for retinol activity equivalents (12.75 and 11.47) and the lowest for energy (0.70 and 0.77) in both studies. Energy and major nutrient ratios were 0.70–2.26 and 0.77–1.86, and micronutrient ratios were 0.80–12.75 and 0.81–11.47, respectively. The intra-individual variance ratios for almost all nutrients were near 1.

**Table 4:** Relative contributions of intra- and inter-individual variance in dietary nutrient intake: comparison between the J-MICC and JPHC-NEXT studies.

	J-MICC			JPHC-NEXT			A/C
	Percentage contributions of variance components			Percentage contributions of variance components			
	Intra-individual (A)	Inter-individual (B)	A/B	Intra-individual (C)	Inter-individual (D)	C/D	
Energy	41.2	58.8	0.70	43.5	56.5	0.77	0.95
Protein	54.7	45.3	1.21	54.3	45.7	1.19	1.01
Lipid	69.3	30.7	2.26	65.0	35.0	1.86	1.07
Carbohydrate	41.4	58.6	0.71	47.6	52.4	0.91	0.87
Sodium	63.7	36.3	1.75	59.3	40.7	1.46	1.07
Potassium	44.4	55.6	0.80	44.9	55.1	0.81	0.99
Calcium	59.3	40.7	1.46	53.7	46.3	1.16	1.10
Iron	57.8	42.2	1.37	55.1	44.9	1.23	1.05
Beta-carotene equivalents	73.1	26.9	2.71	76.8	23.2	3.31	0.95
Retinol activity equivalents	92.7	7.3	12.75	92.0	8.0	11.47	1.01
Vitamin D	87.4	12.6	6.93	85.0	15.0	5.67	1.03
α-Tocopherols	76.6	23.4	3.27	64.6	35.4	1.83	1.19
Vitamin B_1_	70.3	29.7	2.37	68.8	31.2	2.21	1.02
Vitamin B_2_	62.6	37.4	1.67	62.7	37.3	1.68	1.00
Folate	60.6	39.4	1.54	54.5	45.5	1.20	1.11
Ascorbic acid	55.9	44.1	1.27	51.6	48.4	1.06	1.08
Saturated fatty acid	71.5	28.5	2.51	66.7	33.3	2.01	1.07
Monounsaturated fatty acid	71.4	28.6	2.50	70.8	29.2	2.42	1.01
Polyunsaturated fatty acid	72.2	27.8	2.60	70.4	29.6	2.38	1.03
Cholesterol	78.9	21.1	3.74	74.0	26.0	2.85	1.07
Soluble dietary fiber	59.6	40.4	1.47	52.6	47.4	1.11	1.13
Insoluble dietary fiber	49.2	50.8	0.97	51.4	48.6	1.06	0.96
Total dietary fiber	47.4	52.6	0.90	48.4	51.6	0.94	0.98

J-MICC, The Japan Multi-Institutional Collaborative Cohort Study; JPHC-NEXT, The Japan Public Health Center-based Prospective Study for the Next Generation.

## Discussion

In this comparison of procedures for WFR, including standardization, under the differing J-MICC and JPHC-NEXT studies, we found that, although the two procedures differed in certain minor respects, they were almost identical. Results suggested that these differences had relatively little impact on study results.

One important factor in improving the quality of WFRs is improving the quality of investigators. Investigators in both studies were trained to a technical level essential for survey standardization. In particular, we emphasize the need to improve methods of estimating weight from serving size and coding. In addition, all investigators were provided repeated training to ensure data quality during the survey.

Differences in WFR procedures included the following. First, survey dates differed with regard to consecutive versus non-consecutive days. Murakami et al.^15^ suggested that two or more non-consecutive days, including a weekend day, would be appropriate for determining personal habitual intake. This was because when the survey is conducted on consecutive days, dish contents become similar to those of the previous day due to the consumption of leftovers from the previous day or foodstuff recycling. In this way a difference in survey dates (consecutive or non-consecutive) might also influence intra-individual variation (day-to-day variance). Preceding studies^16^, ^17^, ^18^ reported intra-class correlation coefficients and absolute differences but did not directly investigate intra-individual and inter-individual variance. Furthermore, domestic studies of the relative contribution of intra-individual and inter-individual variance indicated that both consecutive day^19^, ^20^, ^21^ and non-consecutive-day surveys^22^, ^23^ tended to show that intra-individual variation made a relatively greater contribution for many calculated items than inter-individual variation. Further, intra-individual variation was associated with a mostly lower variation among major nutrients and higher variation among micronutrients. These previous findings are similar to those of our present study. We therefore suggest that the setting of survey dates on either consecutive or non-consecutive days did not significantly influence the results.

We also saw differences between the WFR of individual units and family units (proportional distribution by member of family). Iwaoka et al.^24^ reported that records using proportional distribution by a family member tended to systematically underestimate results compared to those using individual records. Given that their subjects were students of nutritionist training colleges and their mothers, it is unclear whether these results would be reflected in the general population. On the basis of this preceding study,^24^ the JPHC-NEXT study also includes proportional distribution by family-unit participation, so the possibility of underestimation among women in the JPHC-NEXT study cannot be excluded. However, as the distribution of portion sizes was similar between men and woman in both studies, we consider that the influence of assessment using individual or per-family recording has no major influence on the evaluation of WFR.

In strict terms, the results of this study have the weakness of not differentiating whether the results were caused by the differences in methods or merely by the difference in study populations. To differentiate, comparison of results for the same person in the same period would be needed, but maintaining the independence of the results of two methods would then become impossible. We therefore compared the outcomes caused by these differences by checking portion sizes (survey sheet recording method) and by assessing intra- and inter-individual variation in nutrient intake (setting of survey days), on the basis that the difference in procedures would influence these items.

## Conclusions

Through comparison of the procedures of WFRs planned and conducted separately in two independent studies, we found a few differences, such as in the setting of survey days and the survey sheet recording method. We then compared the effect on subsequent outcomes of these differences by checking portion sizes (survey sheet recording method) and intra- and inter-individual variation in nutrient intake (setting of survey days). We found that subsequent outcomes were similar, regardless of these differences in procedure.

## Conflicts of interest

None declared.

## Appendix A Supplementary data

eTable 1. Cumulative % contribution of the top 30 foods for energy assessed using dietary record for 12 days.

eTable 2. Cumulative % contribution of the top 30 foods for carbohydrate assessed using dietary record for 12 days.

eTable 3. Cumulative % contribution of the top 30 foods for protein assessed using dietary record for 12 days.

eTable 4. Cumulative % contribution of the top 30 foods for lipid assessed using dietary record for 12 days.

eTable 5. Cumulative % contribution of the top 30 foods for sodium assessed using dietary record for 12 days.

eTable 6. Cumulative % contribution of the top 30 foods for potassium assessed using dietary record for 12 days.
