# Supplementary material for: Comparison of weighed food record procedures for the reference methods in two validation studies of food frequency questionnaires
Source: J Epidemiol. 2017 Mar 13;27(7):331–7. doi: 10.1016/j.je.2016.08.008 (PMC5498406; doi:10.1016/j.je.2016.08.008)
Supplement: Supplementary file 1 [file mmc1.pdf]

eTable 1. Cumulative % contribution of the top 30 foods for energy assessed using dietary record for 12 days

Food code[a]	Food name[a]	J-MICC			JPHC-NEXT		
		rank	kcal/day	Percent	rank	kcal/day	Percent
1088	Rice/Cooked paddy rice/Well-milled rice	1	496.0	24.5	1	477.4	23.8
14006	Vegetable fats and oils/Vegetable oil, blend	2	83.0	4.1	5	35.8	1.8
1026	Breads/White table bread	3	64.0	3.2	4	36.4	1.8
12004	Hen`s eggs/whole Raw	4	58.0	2.9	2	50.9	2.5
13003	Ordinary liquid milk	5	44.0	2.2	3	41.5	2.1
1064	Macaroni, spaghetti/Dry form/boiled	6	28.0	1.4	22	15.0	0.7
16006	Fermented alcoholic beverages/Beer/Pale	7	28.0	1.4	6	28.0	1.4
11221	Chicken/ broiler meats/Thigh, with skin, raw	8	25.0	1.3	17	18.4	0.9
3003	Sugars/Soft sugars /White	9	24.0	1.2	18	17.1	0.9
17043	Dressings/Mayonnaise/egg yolk type	10	23.0	1.1	26	13.3	0.7
11186	Pork/Sausages/Vienna	11	20.0	1.0	19	16.4	0.8
11123	Pork/large type breeds/Loin, lean and fat, raw	12	19.0	1.0	16	19.3	1.0
1039	Udon/Wet form/boiled	13	18.0	0.9	23	14.8	0.7
7107	Bananas/Raw	14	17.0	0.9	28	12.9	0.6
1048	Chinese noodles/wet form boiled	15	17.0	0.8	12	22.9	1.1
2017	Potatoes/Tuber, raw	16	16.0	0.8	20	16.2	0.8
16001	Fermented alcoholic beverages/Sake jozen	17	16.0	0.8	11	23.4	1.2
11129	Pork/large type breeds/Belly, lean and fat, raw	18	15.0	0.7	15	19.3	1.0
4040	Tofu, Abura-age/Abura-age	19	14.0	0.7	25	13.4	0.7
4046	Natto/Itohiki-natto	20	14.0	0.7	13	20.1	1.0
16015	Distilled alcoholic beverages/Shochu/25%alchol	21	14.0	0.7	10	23.6	1.2
11163	Pork/Ground meat/Raw	22	14.0	0.7	24	13.6	0.7
16009	Fermented alcoholic beverages/Happoshu	23	13.0	0.6	9	23.7	1.2
1015	Wheat flour/Soft flour/first grade	24	13.0	0.6	-	-	-
13025	Yogurt/Whole milk, unsweetened	25	13.0	0.6	-	-	-
11130	Pork/large type breeds/Inside ham, lean and fat, raw	26	12.0	0.6	-	-	-
1085	Rice/Cooked paddy rice,/Brown rice	27	12.0	0.6	27	13.1	0.7
1031	Breads/French bread	28	11.0	0.6	-	-	-
11030	Beefs/dairy fattened steer/Chuck, lean meat, raw	29	11.0	0.6	-	-	-
6153	Onions/Bulb, raw	30	11.0	0.5	-	-	-
14008	Vegetable fats and oils/Rapeseed oil	-	-	-	7	26.0	1.3
1087	Rice/Cooked paddy rice/Under-milled rice	-	-	-	8	24.8	1.2
17045	Miso/Rice-koji miso/light yellow type	-	-	-	14	19.5	1.0
4032	Tofu, Abura-age/Momen-tofu (regular tofu)	-	-	-	21	15.6	0.8
7148	Apples/Raw	-	-	-	29	11.9	0.6
10139	Chum salmon/Shiozake	-	-	-	30	10.8	0.5
	**Cumulative% contribution of the top30 foods for energy**			57.5			54.5

J-MICC, The Japan Multi-Institutional Collaborative Cohort Study ; JPHC-NEXT, The Japan Public Health Center-based Prospective Study for the Next Generation. [a]Food codes and Food name correspond to those of the Standard Tables of Food Composition in Japan 2010

When calculated within the limits of definition age of the J-MICC study, the results were similar (data not shown).

eTable 2. Cumulative % contribution of the top 30 foods for carbohydrate assessed using dietary record for 12 days

Food code[a]	Food name[a]	J-MICC rank	J-MICC g/day	J-MICC Percent	JPHC-NEXT rank	JPHC-NEXT g/day	JPHC-NEXT Percent
1088	Rice/Cooked paddy rice/Well-milled rice	1	109.4	40.5	1	105.4	39.0
1026	Breads/White table bread	2	11.3	4.2	2	6.4	2.4
3003	Sugars/Soft sugars/White	3	6.1	2.3	5	4.4	1.6
1064	Macaroni, spaghetti/Dry form/boiled	4	5.3	2.0	11	2.9	1.1
7107	Bananas/Raw	5	4.6	1.7	7	3.4	1.2
1039	Udon/Wet form/boiled	6	3.8	1.4	9	3.1	1.1
2017	Potatoes/Tuber, raw	7	3.8	1.4	6	3.8	1.4
1048	Chinese noodles/wet form boiled	8	3.3	1.2	4	4.5	1.7
13003	Ordinary liquid milk	9	3.2	1.2	10	3.0	1.1
1015	Wheat flour/Soft flour/first grade	10	2.6	1.0	20	1.9	0.7
6153	Onions/Bulb, raw	11	2.6	1.0	13	2.5	0.9
1085	Rice/Cooked paddy rice/Brown rice	12	2.5	0.9	12	2.8	1.0
1031	Breads/French bread	13	2.4	0.9	-	-	-
16006	Fermented alcoholic beverages/Beer/Pale	14	2.2	0.8	15	2.2	0.8
7148	Apples/Raw	15	2.0	0.8	8	3.2	1.2
1044	Somen and Hiyamugi/Dry form boiled	16	2.0	0.7	26	1.4	0.5
1028	Breads/Bread type rolls	17	1.7	0.6	-	-	-
1128	Buckwheat noodles/Wet form, boiled	18	1.7	0.6	17	2.0	0.8
1086	Rice/Cooked paddy rice,/Half-milled rice	19	1.7	0.6	-	-	-
1049	Steamed Chinese noodles	20	1.6	0.6	16	2.1	0.8
6048	Pumpkin/European (Cucurbita maxima) Fruit, raw	21	1.6	0.6	18	2.0	0.7
13026	Yogurt/Skimmed, sweetened	22	1.6	0.6	21	1.9	0.7
1034	Breads/Soft rolls	23	1.4	0.5	-	0.9	-
6061	Cabbage/Head, raw	24	1.4	0.5	23	1.6	0.6
2006	Sweet potatoes/Tuberous root, raw	25	1.4	0.5	22	1.7	0.6
17007	Shoyu: soy sauces/Koikuchi-shoyu (Common type)	26	1.4	0.5	-	-	-
1111	Nonglutinous rice products/Rice ball	27	1.3	0.5	-	-	-
1117	Glutinous rice products/Rice cake	28	1.3	0.5	25	1.4	0.5
15098	Biscuits/Biscuits/soft	29	1.2	0.4	-	-	-
16047	Coffee/Coffee drink	30	1.2	0.4	-	-	-
1087	Rice/Cooked paddy rice,/Under-milled rice	-	-	-	3	5.4	2.0
17045	Miso/Rice-koji miso/light yellow type	-	-	-	14	2.2	0.8
16009	Fermented alcoholic beverages/Happoshu	-	-	-	19	1.9	0.7
15060	Traditional dry confectioneries/ Rice crackers/Shio-senbei	-	-	-	24	1.6	0.6
6182	Tomatoes/Fruit, raw	-	-	-	27	1.3	0.5
7027	Satsuma mandarins/Segments, normal ripening type, raw	-	-	-	28	1.3	0.5
6214	Carrots/Root without skin(European type), raw	-	-	-	29	1.2	0.5
6134	Japanese radish/ Root without skin, raw	-	-	-	30	1.2	0.5
	**Cumulative% contribution of the top30 foods for carbohydrate**			69.5			66.5

J-MICC, The Japan Multi-Institutional Collaborative Cohort Study ; JPHC-NEXT, The Japan Public Health Center-based Prospective Study for the Next Generation. [a]Food codes and Food name correspond to those of the Standard Tables of Food Composition in Japan 2010

When calculated within the limits of definition age of the J-MICC study, the results were similar (data not shown).

eTable 3. Cumulative % contribution of the top 30 foods for protein assessed using dietary record for 12 days

Food code[a]	Food name[a]	J-MICC			JPHC-NEXT		
		rank	g/day	Percent	rank	g/day	Percent
1088	Rice/Cooked paddy rice/Well-milled rice	1	7.4	10.0	1	7.1	9.4
12004	Hen`s eggs/whole Raw	2	4.7	6.4	2	4.1	5.5
1026	Breads/White table bread	3	2.3	3.1	8	1.3	1.7
13003	Ordinary liquid milk	4	2.2	2.9	3	2.0	2.7
11221	Chicken/ broiler meats/Thigh, with skin, raw	5	2.1	2.8	5	1.5	2.0
11123	Pork/large type breeds/Loin, lean and fat, raw	6	1.4	1.9	7	1.4	1.9
11130	Pork/large type breeds/Inside ham, lean and fat, raw	7	1.4	1.8	14	0.9	1.1
11163	Pork/Ground meat/Raw	8	1.2	1.6	11	1.1	1.5
4046	Natto/Itohiki-natto	9	1.2	1.6	4	1.7	2.2
17007	Shoyu: soy sauces/Koikuchi-shoyu (Common type)	10	1.0	1.4	15	0.9	1.1
1064	Macaroni, spaghetti/Dry form/boiled	11	1.0	1.3	-	-	-
11186	Pork/Sausages/Vienna	12	0.8	1.1	23	0.7	0.9
10154	Mackerel/Raw	13	0.8	1.0	-	-	-
4032	Tofu, Abura-age/Momen-tofu (regular tofu)	14	0.8	1.0	6	1.4	1.9
11030	Beefs/dairy fattened steer/Chuck, lean meat, raw	15	0.7	1.0	-	-	-
13025	Yogurt/Whole milk, unsweetened	16	0.7	1.0	29	0.6	0.8
11089	Beefs/Ground meat/Raw	17	0.7	1.0	-	-	-
4040	Tofu, Abura-age/Abura-age	18	0.7	0.9	27	0.6	0.9
10134	Chum salmon/Raw	19	0.7	0.9	12	1.0	1.3
17045	Miso/Rice-koji miso/light yellow type	20	0.7	0.9	9	1.3	1.7
11219	Chicken/broiler meats/Breast, with skin, raw	21	0.7	0.9	20	0.7	0.9
10241	Yellowtail/Mature, raw	22	0.6	0.8	30	0.6	0.8
13040	Processed cheese	23	0.6	0.8	25	0.7	0.9
4034	Tofu, Abura-age/Soft-tofu	24	0.6	0.8	-	-	-
10003	Horse mackerel/Raw	25	0.6	0.8	-	-	-
13026	Yogurt/Skimmed, sweetened	26	0.6	0.8	22	0.7	0.9
1048	Chinese noodles/wet form, boiled	27	0.6	0.8	18	0.8	1.0
10259	Big-eye tuna/Raw	28	0.6	0.8	13	0.9	1.2
11129	Pork/large type breeds/Belly, lean and fat, raw	29	0.5	0.7	19	0.7	0.9
10345	Japanese common squid/Raw	30	0.5	0.7	16	0.8	1.1
10139	Chum salmon/Shiozake	-	-	-	10	1.2	1.6
4033	Tofu, Abura-age/Kinugoshi-tofu (Tofu with whey)	-	-	-	17	0.8	1.0
11115	Pork/large type breeds/Picnic shoulder, lean and fat, raw	-	-	-	21	0.7	0.9
11176	Pork/Hams/Loin	-	-	-	24	0.7	0.9
16037	Teas/Sencha/infusion	-	-	-	26	0.7	0.9
10173	Pacific saury/Raw	-	-	-	28	0.6	0.8
	**Cumulative% contribution of the top30 foods for protein**			51.6			50.6

J-MICC, The Japan Multi-Institutional Collaborative Cohort Study ; JPHC-NEXT, The Japan Public Health Center-based Prospective Study for the Next Generation. [a]Food codes and Food name correspond to those of the Standard Tables of Food Composition in Japan 2010

When calculated within the limits of definition age of the J-MICC study, the results were similar (data not shown).

eTable 4. Cumulative % contribution of the top 30 foods for lipid assessed using dietary record for 12 days

Food code[a,b]	Food name[a,b]	J-MICC rank	J-MICC g/day	J-MICC Percent	JPHC-NEXT rank	JPHC-NEXT g/day	JPHC-NEXT Percent
14006	Vegetable fats and oils/Vegetable oil, blend	1	9.0	14.5	1	3.9	6.8
12004	Hen`s eggs/whole Raw	2	4.0	6.4	2	3.5	6.0
17043	Dressings/Mayonnaise/egg yolk type	3	2.5	4.0	7	1.4	2.5
13003	Ordinary liquid milk	4	2.5	4.0	4	2.4	4.1
11221	Chicken/ broiler meats/Thigh, with skin raw	5	1.8	2.9	9	1.3	2.2
11186	Pork/Sausages/Vienna	6	1.8	2.8	6	1.5	2.5
11123	Pork/large type breeds/Loin, lean and fat, raw	7	1.4	2.3	8	1.4	2.5
11129	Pork/large type breeds/Belly, lean and fat, raw	8	1.3	2.1	5	1.7	3.0
4040	Tofu, Abura-age/Abura-age	9	1.2	1.9	10	1.2	2.0
1026	Breads/White table bread	10	1.1	1.7	22	0.6	1.1
14001	Vegetable fats and oils/Olive oil	11	1.0	1.7	12	1.0	1.7
11163	Pork/Ground meat/Raw	12	0.9	1.5	13	0.9	1.6
14017	Butters/Salted butter	13	0.9	1.5	19	0.7	1.1
1088	Rice/Cooked paddy rice/Well-milled rice	14	0.9	1.4	15	0.9	1.5
11030	Beefs/dairy fattened steer/Chuck, lean meat, raw	15	0.9	1.4	-	-	-
11046	Beefs/dairy fattened steer/Flank or short plate, lean and fat, raw	16	0.8	1.3	-	-	-
17040	Dressings/French dressing	17	0.8	1.3	-	-	-
11183	Pork/Bacons/Bacon	18	0.8	1.2	17	0.8	1.4
4046	Natto/Itohiki-natto	19	0.7	1.1	11	1.0	1.7
13040	Processed cheese	20	0.7	1.1	18	0.8	1.3
17051	Roux/Curry roux	21	0.7	1.1	24	0.6	1.0
11130	Pork/large type breeds/Inside ham, lean and fat, raw	22	0.7	1.1	-	-	-
14021	Margarines/Fat spread	23	0.7	1.1	-	-	-
13025	Yogurt/Whole milk, unsweetened	24	0.6	1.0	27	0.5	0.9
14002	Vegetable fats and oils/Sesame oil	25	0.6	0.9	-	-	-
11089	Beef/Ground meat/Raw	26	0.6	0.9	-	-	-
15098	Biscuits/Biscuits/soft	27	0.5	0.9	-	-	-
11034	Beefs/dairy fattened steer/Chuck loin, lean and fat, raw	28	0.5	0.8	-	-	-
10241	Yellowtail/Mature, raw	29	0.5	0.8	28	0.5	0.8
4032	Tofu, Abura-age/Momen-tofu (regular tofu)	30	0.5	0.8	14	0.9	1.6
14008	Vegetable fats and oils/Rapeseed oil	-	-	-	3	2.8	4.9
10173	Pacific saury/Raw	-	-	-	16	0.8	1.5
15116	Chocolates/Milk chocolates	-	-	-	20	0.6	1.1
17045	Miso/Rice-koji miso/light yellow type	-	-	-	21	0.6	1.1
10139	Chum salmon/Shiozake	-	-	-	23	0.6	1.0
11176	Pork/Hams/Loin	-	-	-	25	0.6	1.0
11115	Pork/large type breeds/Picnic shoulder, lean and fat, raw	-	-	-	26	0.6	1.0
4033	Tofu, Abura-age/Kinugoshi-tofu (Tofu with whey)	-	-	-	29	0.5	0.8
19322[b]	Dressings Sesame seeds dressing[b]	-	-	-	30	0.4	0.8
	**Cumulative%contribution of the top30 foods for fat**			65.5			60.5

J-MICC, The Japan Multi-Institutional Collaborative Cohort Study ; JPHC-NEXT, The Japan Public Health Center-based Prospective Study for the Next Generation. [a]Food codes and Food name correspond to those of the Standard Tables of Food

Composition in Japan 2010

[b]Food codes and Food name correspond to National Health and Nutrition Survey program "shokuji shirabe"

When calculated within the limits of definition age of the J-MICC study, the results were similar (data not shown).

eTable 5. Cumulative % contribution of the top 30 foods for sodium assessed using dietary record for 12 days

Food code[a,b]	Food_name[a,b]	J-MICC			JPHC-NEXT		
		rank	mg/day	Percent	rank	mg/day	Percent
17007	Shoyu: soy sauces/Koikuchi-shoyu (Common type)	1	775.6	19.8	1	629.4	15.3
17012	Edible salts/Common salt/Shokuen	2	579.4	14.8	2	549.8	13.3
17045	Miso/Rice-koji miso/light yellow type	3	258.6	6.6	3	498.9	12.1
1026	Breads/White table bread	4	124.3	3.2	7	69.0	1.7
17029	Soup stocks/Mentsuyu/ Straight	5	112.9	2.9	14	37.3	0.9
17028	Soup stocks/Seasoning mix, granule	6	94.0	2.4	5	136.4	3.3
17027	Soup stocks/Consomme, cubes	7	89.1	2.3	9	52.1	1.3
7022	Japanese apricots/Umeboshi, salted pickles	8	85.6	2.2	8	56.5	1.4
17051	Roux/Curry roux	9	83.8	2.1	6	72.7	1.8
12004	Hen`s eggs/whole Raw	10	60.6	1.5	10	47.2	1.1
17046	Miso/Rice- koji miso/dark yellow type	11	53.2	1.4	23	19.6	0.5
11186	Pork/Sausages/Vienna	12	46.2	1.2	15	37.2	0.9
17030	Soup stocks/Mentsuyu/Triple strength	13	43.9	1.1	4	231.2	5.6
17001	Seasoning/Worcester sauces/common type	14	41.4	1.1	-	-	-
19402[b]	Citrus flavor soy sauce[b]	15	41.2	1.1	11	42.4	1.0
17008	Shoyu: soy sauces/Usukuchi-shoyu (Light color type)	16	38.2	1.0	19	25.2	0.6
17039	Dressings/Japanese style dressing	17	35.6	0.9	17	25.8	0.6
17043	Dressings/Mayonnaise/egg yolk type	18	31.4	0.8	27	17.9	0.4
1064	Macaroni, spaghetti/Dry form/boiled	19	29.6	0.8	29	17.1	0.4
19405[b]	Japanese barbecue sauce[b]	20	29.6	0.8	21	20.6	0.5
13040	Processed cheese	21	29.3	0.7	16	32.1	0.8
11176	Pork/Hams/Loin	22	28.4	0.7	12	40.6	1.0
17036	Tomato processed foods/Ketchup	23	26.2	0.7	-	-	-
10381	Fish paste products/Yaki-chikuwa	24	25.9	0.7	20	21.3	0.5
1031	Breads/French bread	25	25.9	0.7	-	-	-
13003	Ordinary liquid milk	26	25.9	0.7	18	25.4	0.6
10386	Fish paste products/Satsuma-age	27	23.0	0.6	22	19.7	0.5
17040	Dressings/French dressing	28	22.1	0.6	-	-	-
19851[b]	Soup for Chinese noodles Dried by frying seasoned[b]	29	21.8	0.6	-	-	-
1039	Udon/Wet form/boiled	30	20.8	0.5	30	16.9	0.4
10139	Chum salmon/Shiozake	-	-	-	13	38.9	0.9
10137	Chum salmon/Aramaki (Mild salted whole body), raw	-	-	-	24	19.6	0.5
6066	Cucumber/Salted pickles	-	-	-	25	19.3	0.5
17013	Edible salts/Common salt/Namien	-	-	-	26	18.8	0.5
1059	Chinese style instant cup noodles/Dried by frying	-	-	-	28	17.4	0.4
	**Cumulative% contribution of the top30 foods for sodium**			74.2			69.3

J-MICC, The Japan Multi-Institutional Collaborative Cohort Study ; JPHC-NEXT, The Japan Public Health Center-based Prospective Study for the Next Generation. [a]Food codes and Food name correspond to those of the Standard Tables of Food Composition in Japan 2010

[b]Food codes and Food name correspond to National Health and Nutrition Survey program "shokuji shirabe"

When calculated within the limits of definition age of the J-MICC study, the results were similar (data not shown).

eTable 6. Cumulative % contribution of the top 30 foods for potassium assessed using dietary record for 12 days

Food code[a]	Food name[a]	J-MICC			JPHC-NEXT		
		rank	mg/day	Percent	rank	mg/day	Percent
13003	Ordinary liquid milk	1	98.6	3.6	1	92.9	3.1
2017	Potatoes/Tuber, raw	2	88.2	3.2	3	87.7	2.9
1088	Rice/Cooked paddy rice/Well-milled rice	3	85.6	3.1	5	82.4	2.7
16045	Coffee/infusion	4	77.1	2.8	6	69.4	2.3
7107	Bananas/Raw	5	73.0	2.6	11	54.0	1.8
6267	Spinach/Leaves, raw	6	71.3	2.6	4	84.3	2.8
16037	Teas/Sencha/infusion	7	64.4	2.3	2	88.5	2.9
6061	Cabbage/Head, raw	8	54.6	2.0	9	62.5	2.1
17007	Shoyu: soy sauces/Koikuchi-shoyu (Common type)	9	53.0	1.9	14	43.1	1.4
12004	Hen`s eggs/whole Raw	10	50.0	1.8	12	43.8	1.5
4046	Natto/Itohiki-natto	11	46.3	1.7	8	66.2	2.2
6153	Onions/Bulb, raw	12	44.3	1.6	15	42.6	1.4
6182	Tomatoes/Fruit, raw	13	40.2	1.5	10	58.5	1.9
6048	Pumpkin/European (Cucurbita maxima) Fruit, raw	14	34.7	1.3	13	43.6	1.4
13025	Yogurt/Whole milk, unsweetened	15	34.3	1.2	23	28.4	0.9
11221	Chicken/ broiler meats/Thigh, with skin, raw	16	34.3	1.2	28	24.9	0.8
6214	Carrots/Root without skin(European type), raw	17	32.1	1.2	19	37.0	1.2
17021	Soup stocks/Katsuo-bushi and kombu extracts	18	32.1	1.2	-	-	-
6134	Japanese radish/ Root without skin, raw	19	31.4	1.1	7	69.2	2.3
6233	Chinese cabbage/Head, raw	20	31.1	1.1	16	42.5	1.4
16046	Coffee/instant coffee	21	27.9	1.0	17	38.9	1.3
6065	Cucumber/Fruit, raw	22	26.7	1.0	25	27.6	0.9
16006	Fermented alcoholic beverages/Beer/Pale	23	23.7	0.9	30	23.8	0.8
1026	Breads/White table bread	24	23.6	0.9	-	-	-
11130	Pork/large type breeds/Inside ham, lean and fat, raw	25	23.2	0.8	-	-	-
11123	Pork/large type breeds/Loin, lean and fat, raw	26	22.9	0.8	-	-	-
2010	Taro/Corm, raw	27	22.1	0.8	24	27.8	0.9
6263	Broccoli/Inflorescence, raw	28	21.0	0.8	27	25.0	0.8
2006	Sweet potatoes/Tuberous root, raw	29	20.8	0.8	26	25.2	0.8
17045	Miso/Rice-koji miso/light yellow type	30	20.1	0.7	18	38.7	1.3
6191	Eggplants/Fruit, raw	-	-	-	20	30.6	1.0
4032	Tofu, Abura-age/Momen-tofu (regular tofu)	-	-	-	21	30.3	1.0
6086	Komatsuna/Leaves, raw	-	-	-	22	29.2	1.0
7148	Apples/Raw	-	-	-	29	24.3	0.8
	**Cumulative% contribution of the top30 foods for potassium**			47.5			48.0

J-MICC, The Japan Multi-Institutional Collaborative Cohort Study ; JPHC-NEXT, The Japan Public Health Center-based Prospective Study for the Next Generation. [a]Food codes and Food name correspond to those of the Standard Tables of Food Composition in Japan 2010

When calculated within the limits of definition age of the J-MICC study, the results were similar (data not shown)
